# Identification of prognostic factors in canine mammary malignant tumours: a multivariable survival study

**DOI:** 10.1186/1746-6148-9-1

**Published:** 2013-01-04

**Authors:** Andreia A Santos, Célia C Lopes, Jorge R Ribeiro, Liliana R Martins, Joana C Santos, Irina F Amorim, Fátima Gärtner, Augusto J Matos

**Affiliations:** 1 Department of Veterinary Clinics of the Biomedical Sciences Institute of Abel Salazar (ICBAS), University of Porto, Largo Professor Abel Salazar, 2, Porto, 4099-003, Portugal; 2 Multidisciplinary Unit for Biomedical Research (UMIB), University of Porto, Largo Professor Abel Salazar, 2, Porto, 4099-003, Portugal; 3 Department of Molecular Pathology and Immunology of the Biomedical Sciences Institute of Abel Salazar (ICBAS), University of Porto, Largo Professor Abel Salazar, 2, Porto, 4099-003, Portugal; 4 Institute of Molecular Pathology and Immunology of the University of Porto (IPATIMUP), R Dr. Roberto Frias, s/n, Porto, 4200-465, Portugal

**Keywords:** Canine, Mammary, Tumours, Prognosis, Multivariable, Survival, Study

## Abstract

**Background:**

Although several histopathological and clinical features of canine mammary gland tumours have been widely studied from a prognostic standpoint, considerable variations in tumour individual biologic behaviour difficult the definition of accurate prognostic factors. It has been suggested that the malignant behaviour of tumours is the end result of several alterations in cellular physiology that culminate in tumour growth and spread. Accordingly, the aim of this study was to determine, using a multivariable model, the independent prognostic value of several immunohistochemically detected tumour-associated molecules, such as MMP-9 and uPA in stromal cells and Ki-67, TIMP-2 and VEGF in cancer cells.

**Results:**

Eighty-five female dogs affected by spontaneous malignant mammary neoplasias were followed up for a 2-year post-operative period. In univariate analysis, tumour characteristics such as size, mode of growth, regional lymph node metastases, tumour cell MIB-1 LI and MMP-9 and uPA expressions in tumour-adjacent fibroblasts, were associated with both survival and disease-free intervals. Histological type and grade were related with overall survival while VEGF and TIMP-2 were not significantly associated with none of the outcome parameters. In multivariable analysis, only a MIB-1 labelling index higher than 40% and a stromal expression of MMP-9 higher than 50% retained significant relationships with poor overall and disease-free survival.

**Conclusions:**

The results of this study indicate that MMP-9 and Ki-67 are independent prognostic markers of canine malignant mammary tumours. Furthermore, the high stromal expressions of uPA and MMP-9 in aggressive tumours suggest that these molecules are potential therapeutic targets in the post-operative treatment of canine mammary cancer.

## Background

Mammary tumours are the most prevalent neoplasms in intact female dogs [1] and it has been described that approximately 40 to 50% are histologically malignant [2]. Mammary cancers have, however, variable biological behaviours, hampering estimates of individual clinical outcomes based solely on their histological and clinical characteristics [3].

Several studies reported that factors such as tumour size [4,5], histological type [6], histological grade [7,8], mode of growth [4,9] and lymph node status [10,11] influence the prognosis of canine malignant mammary tumours (MMTs) and these factors are currently used in practice to establish a prognosis. These clinical and histological factors are, however, crude determinants and are not used, in routine practice, as indicators of the need for adjuvant post-operative therapies.

In human breast cancer, molecular markers (oestrogen and progesterone receptors and c-erbB2) are routinely used for prognostic and predictive purposes [12]. In veterinary medicine, although some potential prognostic biomarkers have been investigated in canine MMTs, such as proliferation markers [9,13], hormone receptors [4,11,14,15] and oncogenes [5,16] none has been adopted in the routine pathological processing of spontaneous malignant tumours. This is due, in part, to the small number of prospective multivariable survival studies that demonstrated their prognostic value. Therefore, the investigation of molecular markers with prognostic and predictive value is still required in order to recognize animals in need for adjuvant therapies, as well as to identify new therapeutic targets.

Previously published studies investigated either the characteristics of the tumour cells or those of the entire tumour population (tumour cells and stroma), seldom considering the tumour-stroma crosstalk by the individual characterization of the distinct cell types. However, it has been demonstrated, in human breast cancer, that the interactions between cancer cells and stroma are critical for tumour growth and invasion [17]. There are increasing evidences that both cancer and stromal cells interact in a coordinated way to facilitate proliferation, invasion and angiogenesis, by remodelling the tumour microenvironment through matrix-associated proteases, such as matrix metalloproteinases and serine proteases, that breakdown basal membranes and proteins of the extracellular matrix [18].

In this context, this study aimed to evaluate the prognostic value of several clinical, histological and molecular features of canine MMTs, including angiogenic factors (VEGF), matrix degrading proteins (uPA and MMP-9), and proliferation markers (Ki-67), in a prospective two-years follow up study. Factors significantly related to outcome, identified in univariate analysis, were included in a multivariable study in order to identify independent prognostic factors that may be adopted in routine practice and constitute potential targets of adjuvant therapies.

## Results

The mean ± SD age of the dogs at the time of surgery was 10.3 ± 2.9 years (range 5–15). Thirteen dogs were spayed before or at the time of surgery. Only 8 had received hormonal therapy for oestrous prevention.

The tumour histological types and the corresponding development of recurrences and/or distant metastases are presented in Table 1.

**Table 1:** Histological classification of CMTs according to World Health Organization and description of tumour types with distant metastases

**Histological Type**	**Tumours**		**Metastasized tumours**	
	**Number**	**%**	**Number**	**%**
Solid carcinomas	30	35.3	8	26.7
Complex carcinomas	20	23.5	4	20.0
Tubulopapillary carcinomas	18	21.2	5	27.7
Carcinosarcomas	9	10.6	3	33.3
Anaplastic carcinomas	2	2.4	1	50.0
Carcinomas in benign tumours	2	2.4	2	100
Mucinous carcinomas	2	2.4	1	50.0
Micropapillary carcinomas	1	1.1	1	100
Spindle cell carcinomas	1	1.1	1	100

Overall, 30% of the dogs developed recurrences or distant metastases and 25.8% died or were euthanized due to this feature during the follow-up period. The mean ± SD survival time for dogs with recurrence/distant metastases was 11.96 ± 7.58 months (range 2–24) and the mean time for the detection of recurrence/metastasis was 5.29 ± 5.68 months (range 1–21). The overall 2-year survival was 48%. Within patients that developed recurrences or distant metastases, 38.5% had been spayed and 25% had been treated in the past with progestagens-based therapy for oestrus control.

Host factors were not associated with disease-free survival (DFS) or overall survival (OS) (Table 2), but the mean DFS and OS tended to decrease in the largest breeds. In spite of the small numbers, neither reproductive status nor hormonal therapy significantly influenced OS and DFS.

**Table 2:** Association between studied variables with disease-free and overall survival times

**Variable**	**Animals**		**Recurrence or distant metastases**		**Mean DFS time in months**	Р*	**Dead or euthanized dogs**		**Mean OS time in months**	Р*
	**N**	**%**	**N**	**%**	**(± SE)**		**N**	**%**	**(± SE)**	
***Total***	85	100	26	30.6	5.3 (5.7)		22	25.8	11.9 (7.6)	
***Animal factors***										
**Weight (kg)**						NS				NS
<10	27	31.8	7	25.9	19.6 (1.6)		6	22.2	21.7 (1.2)	
10-23	39	45.9	11	28.2	18.3 (1.5)		10	25.6	20.0 (1.2)	
>23	19	22.3	8	42.1	15.8 (2.2)		6	31.6	19.2 (1.8)	
**Spayed**						NS				NS
No	72	84.7	21	29.2	18.4 (1.1)		17	23.6	20.7 (0.8)	
Yes	13	15.3	5	38.5	16.8 (2.7)		5	38.5	19.3 (1.9)	
**Hormonal therapy**^**b**^						NS				NS
No	74	90.2	23	31.1	18.0 (1.1)		19	25.7	20.5 (0.8)	
Yes	8	9.8	2	25.0	19.5 (2.9)		2	25.0	20.7 (2.4)	
***Tumour factors***										
**Ulceration**						NS				NS
No	74	87.1	21	28.4	18.6 (1.0)		17	23.0	21.0 (0.8)	
Yes	11	12.9	5	45.5	14.6 (3.2)		5	45.5	16.6 (2.8)	
**Size**						0.027				0.016
< 3 cm	47	55.3	10	21.3	20.3 (1.1)		8	17.0	22.1 (0.8)	
≥ 3 cm	38	44.7	16	42.1	15.5 (1.7)		14	36.8	18.3 (1.3)	
**Necrosis**						NS				NS
No	25	29.4	7	28.0	18.3 (1.9)		7	28.0	20.1 (1.5)	
Yes	60	70.6	19	31.2	18.0 (1.2)		15	25.0	20.5 (0.9)	
**Squamous metaplasia**						NS				NS
No	62	72.9	19	30.1	18.1 (1.2)		17	27.4	20.5 (0.9)	
Yes	23	27.1	7	30.4	18.1 (1.9)		5	21.7	20.4 (1.6)	
**Mode of growth**						0.007				0.022
Expansive	24	28.2	2	8.3	22.6 (0.9)		2	8.3	23.3 (0.7)	
Invasive	61	71.8	24	39.3	16.4 (1.3)		20	32.8	19.3 (0.9)	
**Histological type**						NS				0.023
Complex carcinomas	20	23.5	4	20.0	20.7 (1.6)		2	10.0	23.6 (0.3)	
Simple carcinomas	51	60.0	15	29.4	18.5 (1.3)		13	25.5	20.6 (0.9)	
Others	14	16.5	7	50.0	13.1 (2.9)		7	50.0	15.4 (2.4)	
**Mitotic index**						NS				NS
0-9/10hpf	36	42.4	10	27.8	18.6 (1.5)		9	25.0	20.9 (1.2)	
10-19/10hpf	25	29.4	6	24.0	18.9 (1.8)		4	16.0	21.2 (1.3)	
≥ 20/10hpf	24	28.2	10	41.7	16.7 (1.9)		9	37.5	19.1 (1.4)	
**Histological grade**						NS				0.025
I + II	58	68.2	14	24.1	19.3 (1.1)		11	19.0	21.8 (0.8)	
III	27	31.8	12	44.4	15.5 (1.9)		11	40.7	17.7 (1.6)	
**RLN metastases**^**a**^						0.017				0.005
No	47	71.2	10	21.3	20.2 (1.1)		8	17.0	22.2 (0.7)	
Yes	19	28.8	9	47.4	14.6 (2.4)		9	47.4	17.3 (2.1)	
***Cancer cells***										
**MIB1 L.I. (%)**						<0.001				<0.001
< 40	47	55.3	7	14.9	21.1 (1.03)		6	12.8	22.8 (0.7)	
≥ 40	38	44.7	19	50.0	14.4 (1.66)		16	42.1	17.5 (1.4)	
**TIMP-2 (%)**						NS				NS
< 25	32	37.6	7	21.9	19.7 (1.5)		5	15.6	21.6 (1.1)	
≥ 25	53	62.4	19	35.8	17.1 (1.3)		17	32.1	19.7 (1.0)	
**VEGF-A (%)**						NS				NS
< 50	47	55.3	14	29.8	18.4 (1.3)		10	21.3	21.1 (0.9)	
≥ 50	38	44.7	12	31.6	17.7 (1.5)		12	31.6	19.7 (1.0)	
***Stromal cells***										
**uPA (%)**						0.016				0.012
< 10	25	29.4	3	12.0	21.9 (1.1)		2	8.0	23.5 (0.4)	
≥ 10	60	70.6	23	38.3	16.5 (1.3)		20	33.3	19.1 (1.0)	
**MMP-9 (%)**						<0.001				<0.001
< 50	54	63.5	9	16.7	20.9 (0.9)		8	14.8	22.5 (0.7)	
≥ 50	31	36.5	17	54.8	13.2 (1.9)		14	45.2	16.7 (1.5)	

^a^ Lymph nodes were not submitted in 19 cases.

^b^ The information regarded hormonal therapy was impossible to obtain in three cases.

*Log Rank test.

NS – not significant.

**Figure 1:**
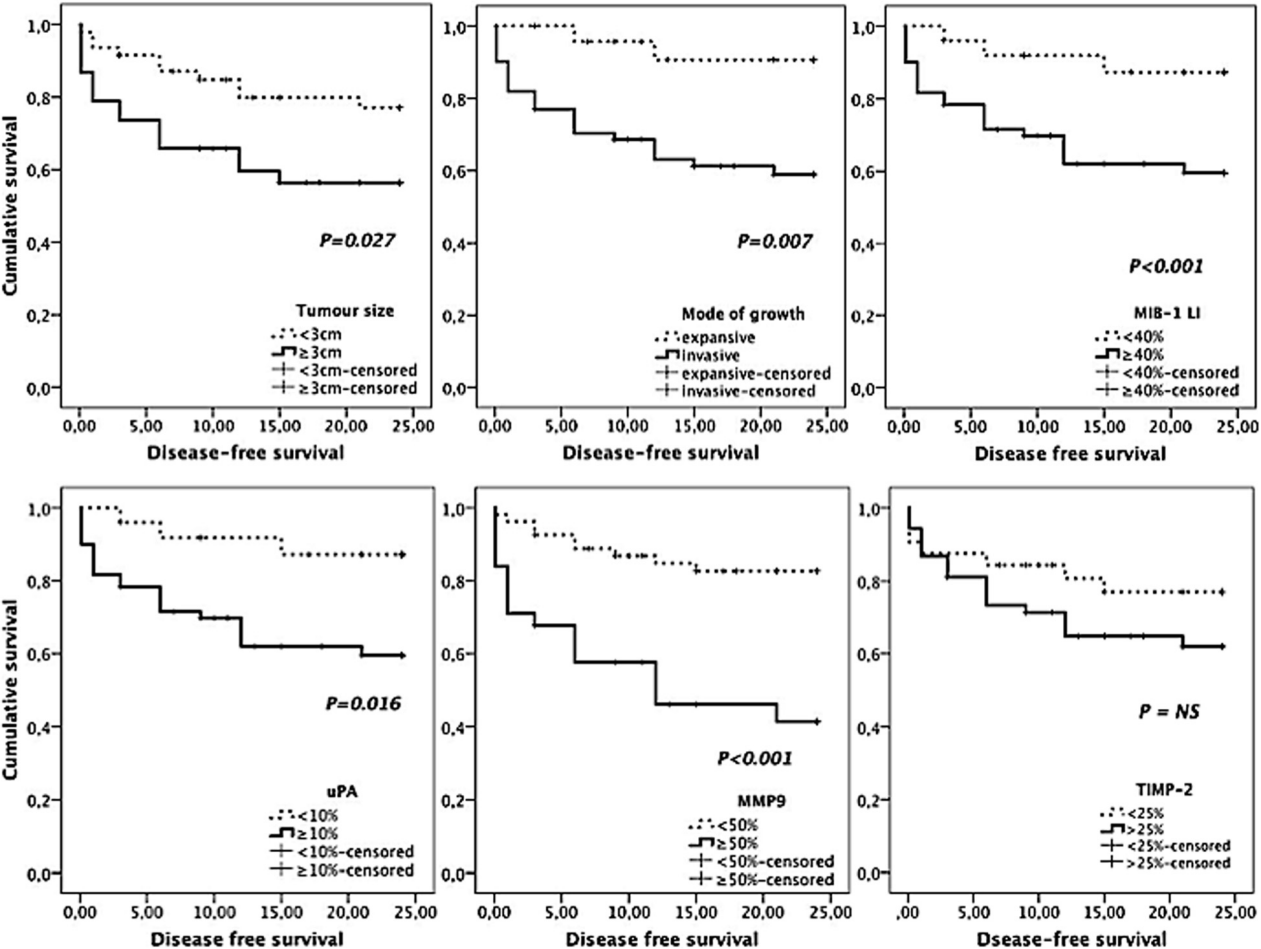
**Kaplan-Meier disease free survival curves comparing immunohistochemical clinico-pathological factors in 85 dogs with MMTs.** Female dogs with tumours larger than 3 cm, invasive growth, MIB-1 labelling index higher than 40%, and high uPA and MMP-9 immunoexpressions in stromal cells had significantly shorter disease-free intervals.

**Figure 2:**
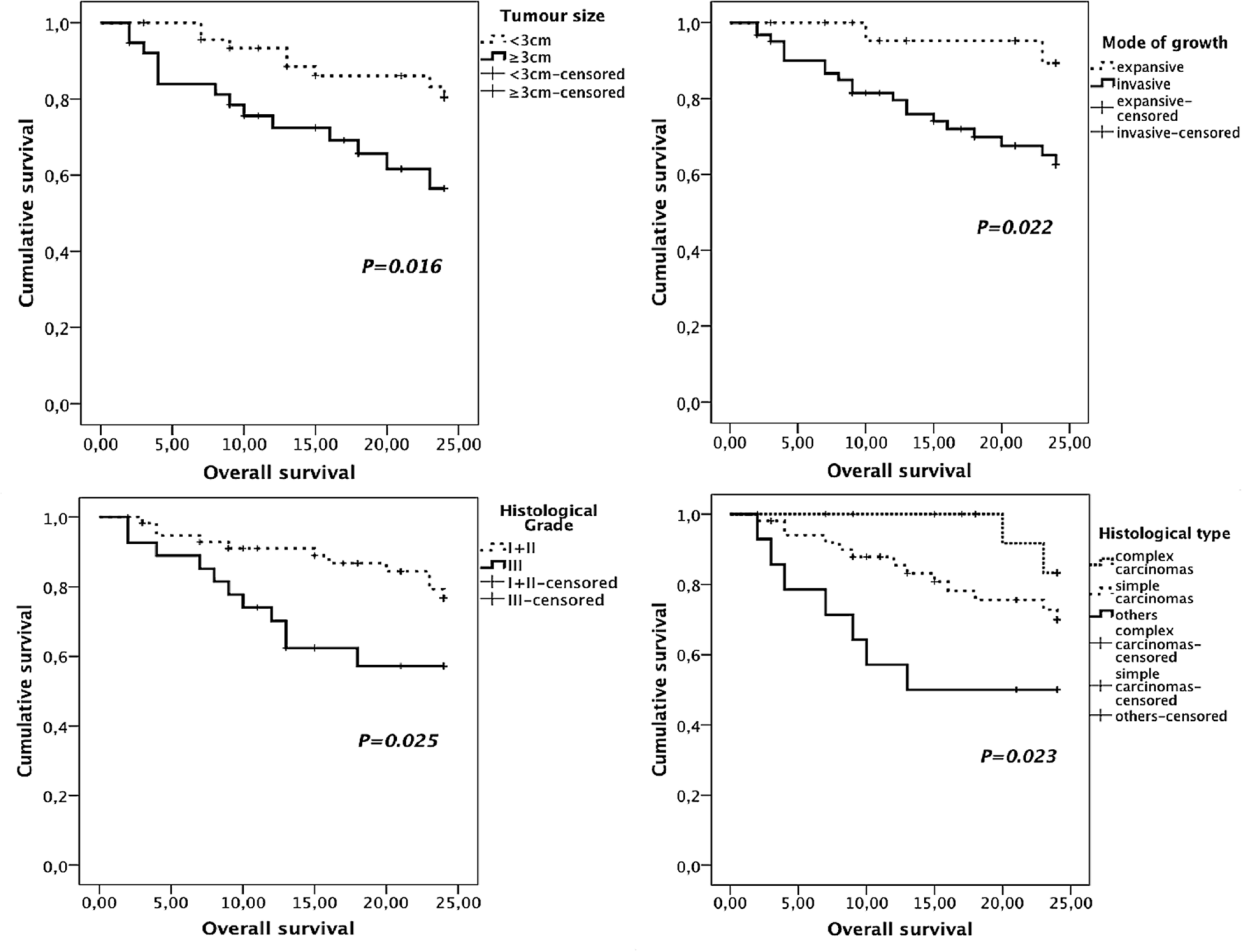
**Kaplan-Meier overall survival curves comparing clinical and histopathological parameters in 85 dogs with MMTs.** Dogs with tumours smaller than 3 cm, with expansive growth, well to moderately differentiated and classified as complex carcinomas had significantly longer survival times.

Animals with complex carcinomas presented longer survival times and lower risk of death caused by MMT, when compared to the “others” group but not to those with simple carcinomas (Tables 2 and 3). No relationships were found between ulceration, necrosis or squamous metaplasia and OS or DFS. Larger tumours (≥ 3 cm) and tumours with invasive growth were associated to shorter DFS (Table 2; Figure 1) and OS (Table 2; Figure 2), an increased risk to the development of recurrences/distant metastases (Table 4) and to cause death due to MMT (Table 3). Grade III tumours were associated with shorter OS (Table 2; Figure 2) and increased risk of related death (Table 3). Mitotic index was not related with patient survival (Table 2). Regional lymph node (RLN) metastases were significantly associated with shorter OS and DFS times (Table 2), increased risk of recurrence/distant metastasis (HR: 2.8; 95% CI: 1.13-6.91; *P* = 0.025) and related death (HR: 3.56; 95% CI: 1.37-9.26; *P* = 0.009).

**Table 3:** Prognostic factors significantly associated with overall survival

**Variable**	**Univariate**			Multivariable*		
	**Hazard ratio**	**95% CI**	***P***	**Hazard ratio**	**95% CI**	***P***
**Tumour size**						
< 3 cm	Referent					
≥ 3 cm	2.79	1.16-6.68	0.022			NS
**Mode of growth**						
Expansive	Referent					
Invasive	4.63	1.08-19.85	0.039			NS
**Histological type**						
Complex carcinomas	Referent					
Simple carcinomas	2.75	0.62-12.17	NS			
Others	6.48	1.34-31.22	0.020			NS
**Histological grade**						
I + II	Referent					
III	2.52	1.09-5.82	0.031			NS
**MIB1 L.I. (%)**						
< 40	Referent			Referent		
≥ 40	4.60	1.79-11.85	0.002	4.06	1.56-10.58	0.004
**uPA (%)**						
< 10	Referent					
≥ 10	5.28	1.23-22.60	0.025			NS
**MMP-9 (%)**						
< 50	Referent			Referent		
≥ 50	4.21	1.76-1.09	0.001	3.67	1.52-8.89	0.004

* Cox proportional hazard model: forward stepwise method – likelihood ratio.

NS – not significant.

**Table 4:** Prognostic factors significantly associated with disease free survival

**Variable**	**Univariate**			Multivariable*		
	**Hazard ratio**	**95% CI**	***P***	**Hazard ratio**	**95% CI**	***P***
**Tumour size**						
< 3 cm	Referent					
≥ 3 cm	2.33	1.06-5.14	0.036			NS
**Mode of growth**						
Expansive	Referent					
Invasive	5.62	1.33-23.80	0.019			NS
**MIB1 L.I. (%)**						
< 40	Referent			Referent		
≥ 40	4.15	1.74-9.90	0.001	3.24	1.35-7.78	0.009
**uPA (%)**						
< 10	Referent					
≥ 10	3.82	1.14-12.72	0.029			NS
**MMP-9 (%)**						
< 50	Referent			Referent		
≥ 50	4.34	1.93-9.77	<0.001	3.38	1.49-7.67	0.004

*Cox regression model: forward stepwise method – likelihood ratio.

NS - not significant.

**Figure 3:**
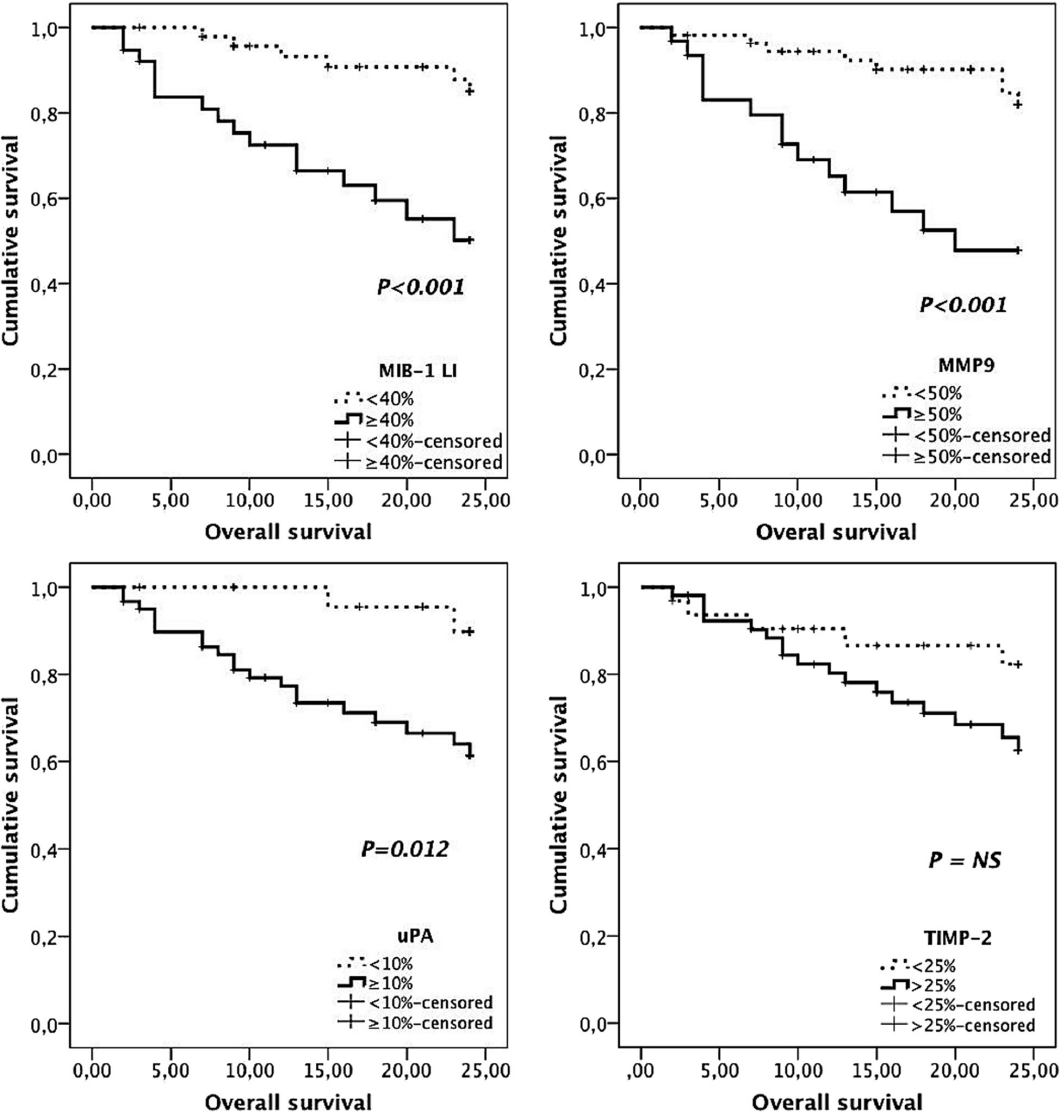
**Kaplan-Meier overall survival curves comparing several immuno-histochemical factors in 85 dogs with MMTs.** MIB-1 labelling index higher than 40%, and increased MMP-9 and uPA immunoexpressions predicted poorer survival times.

The mean ± SD MIB-1 labelling index (LI) was 39.6 ± 2.0 percent (range 6.5- 84.2). Factors significantly related to shorter DFS, shorter OS, tumour-related death and higher risk of recurrences/distant metastases included high MIB-1 LI and high uPA and MMP-9 expressions by tumour-adjacent fibroblasts (Tables 2 and 3; Figures 1 and 3). VEGF and TIMP-2 expressions were not associated with DFS or OS (Table 2).

Multivariable analysis demonstrated that high MIB-1 LI (≥40%) and high MMP-9 expression by stromal fibroblasts (≥ 50%) were the only factors that retained statistical significance as independent predictors of shorter DFS (Table 4) and OS (Table 3).

## Discussion

In this study host related factors such as weight, reproductive status and hormonal therapies were not significantly related to patient outcomes. Although these conclusions are in agreement with previous studies [4,10,19,20], the lack of influence of the reproductive status and hormonal therapies must be regarded with caution since only six animals were spayed at the time of mastectomy and only eight had received oestrous control therapy.

Some prognostic studies suggested that there is an increasing malignancy from complex carcinomas to simple carcinomas to sarcomas [3,6,16,21], although this fact was not demonstrated in other publications [4,20,22]. In this study, considering the most frequent histological types (number > 5), carcinosarcomas were the most aggressive tumours (33% local recurrences and/or distant metastases), while complex carcinomas were the less aggressive ones (20% local recurrences and/or distant metastases and significantly lower risk of death due to MMTs). The differences between simple carcinomas and the other groups were not significant, demonstrating that solid and tubulopapillary carcinomas are probably an heterogeneous group of neoplasms, with distinct invasive and metastatic capacities equally distributed among both groups, as demonstrated by the very similar percentages of local recurrences and/or distant metastases (26.7% for solid and 27.7% for tubulopapillary carcinomas).

Ulceration and necrosis, two features that have been suggested to be indicators of higher tumour aggressiveness [4,6,10], were not significantly related to prognosis in this study. Although ulceration may be caused by the tumour invasive growth, it must be highlighted that it may also be due to self-induced trauma, skin ischemia or infection, features that are not necessarily associated to an aggressive biological behaviour. In a previous study [4], necrosis was associated to poorer outcome but different assessment methodologies may explain the divergent results.

Although squamous metaplasia is often regarded as a sign of tumour aggressiveness in human breast cancer [23,24], this is the first study in canine mammary tumours (CMTs) to address it as an independent variable and, contradicting the previous notion, squamous metaplasia failed to demonstrate a significant prognostic value.

Corroborating previous findings [2,7,10,15], our study showed that histological grade might be helpful to predict survival time, although not time to recurrence or metastasis. It must be remembered, however, that several grading methods have been used to classify MMTs in dogs. The Nottingham method is, in the authors’ opinion, a well standardized method that, although developed for human breast cancer, is applicable to canine MMTs and, as these results demonstrate, associated to survival time.

High MIB-1 LI were strongly associated to the development of recurrences/distant metastases as well as with shorter DFS and OS intervals, both in univariate and multivariable analysis. Half of the animals bearing tumours with LI higher than 40% developed local recurrences and/or distant metastasis and 42% died within 2 years after surgery. Previous CMTs studies associated higher MIB-1 LI with other aggressive tumour features (larger size [5,13], infiltrative growth [6], high histological grade [13]), higher risk of metastatic disease [11] and decreased DFS and OS [7,9], strengthening our findings. Other multivariable studies, however, reported opposite results [9,22,25], which may be explained by the small number of cases studied in the Lee et al. (2004) [22] series and by the different assessment methodologies between studies. Our methodology consisted in the evaluation of the highest labelled areas. When compared to the previously described counting in 3–5 randomized fields [22], our method eases the work of the observer, reduces variability between samples, and allows a more rigorous comparison between large tumours, where it is easier to find suitable non-overlapping fields, and smaller ones, where the choice of fields is more limited. Mitotic index, frequently used to assess tumour proliferative activity, was not associated to patient prognosis in this series. Therefore, it seems that MIB-1 it is a more suitable proliferation-associated prognostic indicator than mitotic index.

The presence of RLN metastases was associated to an increased risk for the development of recurrence or distant metastases and tumour-associated death in univariate analysis. Unfortunately, it was not possible to demonstrate its value as an independent prognostic factor in multivariable analysis because RLN were not surgically removed in 19 (22%) cases. Apparently, as demonstrated by previous studies, this variable fails to maintain its prognostic significance when included in multivariable models [4,6].

In human breast cancer studies, it has been proposed that the expression of VEGF by cancer cells is a poor prognostic factor for survival [26], but this hypothesis was contested by other studies [27,28]. To the best of our knowledge, there is only one previous CMTs survival study that addressed VEGF expression [29] and reported it not to be associated with OS. Our findings demonstrate that VEGF expression by cancer cells is not associated with either OS or DFS, suggesting that this angiogenic factor is not useful as a prognosticator of canine MMTs. Recently, Al-Dissi et al. (2010) [30] verified that there were no correlations between the expression of VEGF, its receptor-2 and tumour microvascular density, suggesting that other factors are more important than VEGF in CMTs angiogenesis. Furthermore, it has been suggested that VEGF could be an early carcinogenic factor that declines with malignant progression [31], a hypothesis that may justify our results.

The protein TIMP-2, initially described as an inhibitor and regulator of the activity of matrix metalloproteinase-2 (MMP-2), has recently been proven to stimulate cell growth and angiogenesis, as well as inhibit apoptosis, hence contributing to tumour aggressiveness [32]. Although no other CMTs study groups assessed the prognostic value of TIMP-2 expression, human breast cancer studies revealed that high levels of TIMP-2 mRNA and TIMP-2 protein are correlated with the development of distant metastasis and decreased DFS [33,34]. In the present study, the quantitative expression of TIMP-2 (i.e. number of positive cells) was not related to outcome. Although this may seem in contradiction with our previous results [35] it must be noticed that the expression of the molecule was evaluated, in that study, using a score obtained by evaluating both number and intensity of expression. However, the reproducibility of intensity scales is very difficult in the routine setting. Therefore, we decided to use a more reproducible classification system in this study.

An increasing number of studies over the last decade identified the tumour stroma as a major player of the carcinogenic process reviewed by [17]. During tumour invasion, cancer cells interact with their microenvironment to activate signalling pathways and to increase growth factors bioavailability, thus favouring tumour progression [18]. Stromal proteases, such as MMPs and uPA, are frequently upregulated in tumour microenvironment and influence tumour behaviour by tissue architecture disruption and signalling interactions [17]. We demonstrated that stromal expressions of both uPA and MMP-9 were associated to poorer outcomes in univariate study, although only MMP-9 was able to maintain its independent prognostic value in multivariable analysis. We also verified that stromal expressions of uPA and MMP-9 are highly associated, which may justify why uPA lost significance in the multivariable model that included MMP-9. To the best of our knowledge, this is the first multivariable survival study addressing these stromal markers in canine MMTs.

## Conclusions

The results of this study demonstrate that the expressions of MMP-9 by stromal cells and of Ki-67 by cancer cells are independent prognostic factors in canine MMTs, that may be used for the selection of those animals that should be considered for post-operative ancillary treatments. These factors exhibited a stronger prognostic value than clinical or histological parameters traditionally used to predict patient’s outcome, such as tumour size, histological grade and mode of growth. Hence, we suggest that they should be considered in the routine prognostic assessment to help practitioners in the prediction of patients’ outcome.

In addition, our findings highlight the role of tumour stroma in the biological behaviour of canine MMTs and suggest that uPA and MMP-9 may be potential targets for post-operative therapies.

## Methods

### Animals and samples

Eighty-five female dogs with spontaneous MMTs underwent surgical treatment and were enrolled, with owners consent, in a 2-year post-operative follow-up study. The study protocol was performed in compliance with the European Union Directives for the protection of animals used for scientific purposes (1999/575/CE and 2010/63/UE) and approved by the Ethics Committee of the Biomedical Sciences Institute of Abel Salazar, University of Porto.

Animal data such as weight, age, reproductive status and previous administration of progestagens for oestrus control, were obtained and registered and, for statistical purposes, dogs were grouped into three categories: small (<10 Kg), medium (10–23 Kg), and large breeds (>23 Kg) according to the criteria of the Féderation Cynologique Internationale (FCI). The clinical staging was assessed in each animal, based in a complete physical examination, three views thoracic radiographies, and a complete abdominal ultrasound evaluation. Inclusion criteria were animals with stage I to IV MMTs whose owners declined post-operative adjuvant therapies. Exclusion criteria were animals with stage V MMTs and animals with a previous history of neoplastic disease.

Removed tumours were fixed in 10% neutral buffered formalin for 48 h., measured in their largest diameter and grouped in two categories: < 3 cm or ≥ 3 cm for statistical purposes. Tumours < 1 cm were paraffin-embedded in one block, while larger tumours were cut sequentially at 5 mm intervals to provide tissue blocks representative of the entire lesion. After dehydration and embedding in paraffin wax, 3 μm sections were obtained from each block. Slides were stained using haematoxylin and eosin (HE) and the histological classification was performed by two pathologists (FG and IA) using the criteria of the World Health Organization for the histological classification of mammary tumours of domestic animals [3]. For statistical purposes, tumours were grouped as: complex carcinomas, simple carcinomas (solid, tubulopapillary, micropapillary and anaplastic), and other (mucinous carcinomas; spindle cell carcinomas; carcinosarcomas and carcinomas in benign tumour). Representative sections were then selected for the immunohistochemical studies.

When available, local and regional lymph nodes were processed and examined as previously described [36]. The mitotic index was calculated and classified as low (less than 10 mitotic figures per 10 hpf); moderate (10 to 19 per 10 hpf); and high (more than 20 per 10 hpf). Histological grading was determined, according to the Nottingham method [37], as Grade I (well differentiated), Grade II (moderately differentiated) and Grade III (poorly differentiated), as previously described in canine mammary tumours [8]. Tumour growth was classified as expansive (cohesive and well delimited mass pushing normal surrounding tissues) or invasive (infiltrative growth or lymphatic or blood vessels invasion). The existence of necrosis and squamous metaplasia, when detected, was registered.

When more than one malignant neoplasm were diagnosed (22 cases, including 4 animals that metastasized), the tumour with the more aggressive clinical and histopathological features (larger size, infiltrative growth, higher grade) was selected.

### Immunohistochemical study

Serial tumour sections were immunostained for Mindbomb homolog 1 (MIB1); VEGF; uPA; and TIMP-2 as described, respectively, by Matos et al. (2006a) [13] and Santos et al. (2010; 2011a,b) [35,38,39]. MMP-9 staining was performed according to a previously described IHC method [39] using the anti-MMP-9 (C-20) goat polyclonal antibody (Santa Cruz Biotechnology) diluted 1:200 in TBS with 5% BSA.

To determine the MIB-1 labelling index, the areas of highest expression were selected and 1000 tumour cells nuclei were counted in these areas at 400× magnification with the help of a microscopic grid. The proportion of stained nuclei was recorded as a percentage [13] and tumours were grouped, for statistical purposes, according to its mean value (40%). Regarding VEGF and TIMP-2 cytoplasmic immunoexpressions, tumours were grouped using cut-off values of 50% and 25% positive cells, respectively. The evaluation of the uPA and MMP-9 expressions was based on the percentage of tumour-associated stromal cells (fibroblasts) with cytoplasmic staining, using cut-off values of 10% for uPA [39] and 50% for MMP-9.

Sections were examined independently by two observers (A.S. and A.M.) and when there was disagreement (less than 5% of the cases) a consensus was reached using a multi-head microscope.

### Follow-up study

Dogs were evaluated before surgery, 3 weeks after surgery and every 3 months thereafter for a 2-year period. Owners were instructed to report and discuss with the researchers any detected abnormalities, even if not obviously related to the mammary gland tumours, at any time. Each evaluation included a thorough physical examination, thoracic radiographs (three views) and complete abdominal ultrasound. Complete necropsies were performed, after owner consent, in all dogs that died spontaneously or were euthanized, and suspected metastases, when present, were histologically confirmed. Overall survival was calculated from the date of surgery to the date of animal death/euthanasia due to tumour metastasis. Disease-free survival was calculated from the date of surgery to the date of detection of the first local recurrence or distant metastases.

### Statistical analysis

Survival curves were calculated using the Kaplan-Meier method and the log-rank test was used to analyse the significance of the differences between the groups defined for each variable [40]. In the OS study, dogs were censored if and when they died for causes unrelated to MMTs, were lost to follow-up, or were alive 24 months after surgery; in the DFS study, dogs were censored if and when they were lost to follow-up, died for causes unrelated to MMTs before developing recurrences or metastases, or were free of distant metastases 24 months after surgery. For each variable, the hazard of recurrence or distant metastasis and the hazard of tumour-related death were estimated by Cox regression analysis [40]. Variables significantly associated with OS or DFS in univariate analyses were included in the multivariable Cox proportional hazards model (forward stepwise method) in order to select the outcome predictors that retained significance, controlling for confounding variables. Due to the high number of missing cases, RLN status was not considered for inclusion on the multivariable model. The significance level was set at *P* < 0.05. Statistical analysis was performed with the statistical package PASW Statistics 18.0.

## Abbreviations

BSA: Bovine serum albumin; CMT: Canine mammary tumour; CI: Confidence interval; DFS: Disease-free survival; HE: Haematoxylin and eosin; HPF: Higher power field; HR: Hazard rate; IHC: Imunohistochemistry; MIB-1 LI: Mindbomb homolog-1 labelling index; MMP: Matrix metalloproteinase; MMT: Malignant mammary tumour; OS: Overall survival; RLN: Regional lymph node; SD: Standard deviation; TIMP-2: Tissue inhibitor of matrix metalloproteinase-2; TBS: Tris-buffered saline; uPA: Urokinase-type plasminogen activator; VEGF: Vascular endothelial growth factor.

## Competing interests

The authors declare that they have no competing interests.

## Authors’ contributions

AS and AM conceived the study, participated in its design and coordination, data aquisition and analysis, and manuscript elaboration. LM, JS, JR participated in data aquisition. AS and CL carried out the immunohistochemistry assay. FG participated in the study design and coordination, performed the histopathological evaluation and helped with the interpretation of data. IA participated in the histopathological evaluation. All authors read and approved the final manuscript.

## References

[B1] Lana SE Rutteman GR Withrow SJ Withrow SJ, MacEwen EG Tumors of the mammary gland Small Animal Clinical Oncology 2007 4th St. Louis: Saunders Elsevier 619 636

[B2] Sorenmo K Canine mammary gland tumors Vet Clin North Am Small Anim Pract 2003 33 573 596 10.1016/S0195-5616(03)00020-2 12852237

[B3] Misdorp W Else RW Helmén E Lipscomb TP Shulman FL Histological classification of the mammary tumours of the dog and the cat World Health Organization International Histological Classification of Tumours of Domestic Animals 1999 2nd Washington DC: Armed Forces Institute of Pathology 16 29

[B4] De Las Mulas J Millán Y Dios R A prospective analysis of immunohistochemically determined estrogen receptor α and progesterone receptor expression and host and tumor factors as predictors of disease-free period in mammary tumors of the Dog Vet Pathol 2005 42 200 212 10.1354/vp.42-2-200 15753474

[B5] Ferreira E Bertagnolli AC Cavalcanti MF Schmitt FC Cassali GD The relationship between tumour size and expression of prognostic markers in benign and malignant canine mammary tumours Vet Comp Oncol 2009 7 230 235 10.1111/j.1476-5829.2009.00193.x 19891693

[B6] Hellmén E Bergstrom R Holmberg L Spangberg IB Hansson K Lindgren A Prognostic factors in canine mammary tumors: a multivariate study of 202 consecutive cases Vet Pathol 1993 30 20 27 10.1177/030098589303000103 8442324

[B7] Peña LL Nieto AI Pérez-Alenza D Cuesta P Castãno M Immunohistochemical detection of Ki-67 and PCNA in canine mammary tumors: relationship to clinical and pathologic variables J Vet Diagn Invest 1998 10 237 246 10.1177/104063879801000303 9683072

[B8] Karayannopoulou M Kaldrymidou E Constantinidis TC Dessiris A Histological grading and prognosis in dogs with mammary carcinomas: application of a human grading method J Comp Pathol 2005 133 1 7 10.1016/j.jcpa.2004.11.006 16202421

[B9] Sarli G Preziosi R Benazzi C Castellani G Marcato PS Prognostic value of histologic stage and proliferative activity in canine malignant mammary tumours J Vet Diagn Invest 2002 14 25 34 10.1177/104063870201400106 12680640

[B10] Pérez-Alenza MD Peña L Nieto I Castaño M Clinical and pathological prognostic factors in canine mammary tumors Ann Ist Super Sanita 1997 33 581 585 9616968

[B11] Nieto A Peña L Pérez-Alenza MD Sánchez MA Flores JM Castãno M Immunohistologic detection of estrogen receptor alpha in canine mammary tumours: clinical and pathologic associations and prognostic significance Vet Pathol 2000 37 239 247 10.1354/vp.37-3-239 10810988

[B12] Walker RA Immunohistochemical markers as predictive tools for breast cancer J Clin Pathol 2008 61 689 696 18037665 10.1136/jcp.2006.041830

[B13] Matos AJF Lopes CCC Faustino AMR Carvalheira JGV Santos MSA Rutteman GR Gärtner MFMR MIB-1 indices according to clinico-pathological variables in canine mammary tumours: a multivariate study Anticancer Res 2006 26 1821 1826 16827113

[B14] Gama A Alves A Schmitt F Identification of molecular phenotypes in canine mammary carcinomas with clinical implications: application of the human classification Virchows Arch 2008 453 123 132 10.1007/s00428-008-0644-3 18677512

[B15] Sassi F Benazzi C Castellani G Sarli G Molecular-based tumour subtypes of canine mammary carcinomas assessed by immunohistochemistry BMC Vet Res 2010 6 5 10.1186/1746-6148-6-5 20109214 PMC2837647

[B16] Hsu W Huang H Liao J Wong M Chang S Increased survival in dogs with malignant mammary tumours overexpressing HER-2 protein and detection of a silent single nucleotide polymorphism in the canine HER-2 gene The Vet Journal 2009 180 116 123 10.1016/j.tvjl.2007.10.013 18061495

[B17] Egeblad M Nakasone ES Werb Z Tumors as organs: complex tissues that interface with the entire organism Develop Cell 2010 18 884 901 10.1016/j.devcel.2010.05.012 PMC2905377 20627072

[B18] Brooks SA Lomax-Browne HJ Carter TM Kinch CE Hall DMS Molecular interactions in cancer cell metastasis Acta Histochem 2010 112 3 25 10.1016/j.acthis.2008.11.022 19162308

[B19] Morris JS Dobson JM Bostock DE O’ Farrell E Effect of ovariohysterectomy in bitches with mammary neoplasms Vet Record 1998 142 656 658 10.1136/vr.142.24.656 9670443

[B20] Philibert JC Snyder PW Glickman N Knapp DW Waters DJ Influence of host factors on survival in dogs with maligant mammary gland tumors J Vet Intern Med 2003 17 102 106 10.1111/j.1939-1676.2003.tb01330.x 12564734

[B21] Yamagi T Kobayashi T Takahashi K Sugiyama M Prognosis for canine malignant mammary tumors based on TNM classification and histologic classification J Vet Med Sci 1996 58 1079 1083 10.1292/jvms.58.11_1079 8959655

[B22] Lee C Kim W Lim J Kang M Kim D Kweon O Mutation and overexpression of p53 as a prognostic factor in canine mammary tumors J Vet Sci 2004 5 63 69 15028887

[B23] Rayson D Adjei AA Suman VJ Wold LE Ingle JN Metaplastic breast cancer: prognosis and response to systemic therapy Annals of Oncol 1999 10 413 419 10.1023/A:1008329910362 10370783

[B24] Tse JM Pan PH Putti TC Lui PCW Chaiwun B Law BKB Metaplastic carcinoma of the breast: clinicopathological review J Clin Pathol 2006 59 1079 1083 10.1136/jcp.2005.030536 16467167 PMC1861754

[B25] Löhr CV Teifke JP Failing K Weiss E Characterization of the proliferation state in canine mammary tumors by the standard AgNOR method with post fixation and immunohistologic detection of Ki-67 and PCNA Vet Pathol 1997 34 212 221 10.1177/030098589703400306 9163877

[B26] Gasparini G Prognostic value of vascular endothelial growth factor in breast cancer Oncolologist 2000 5 suppl 1 37 44 10.1634/theoncologist.5-suppl_1-37 10804090

[B27] Mylona E Alexandrou P Giannopoulou I Liapis G Sofia M Keramopoulos A Nakopoulou L The prognostic value of vascular endothelial growth factors (VEGFs)-A and –B and their receptor, VEGFR-1, in invasive breast carcinoma Gynecol Oncol 2007 104 557 563 10.1016/j.ygyno.2006.09.031 17150246

[B28] Nieto Y Woods J Nawaz F Baron A Jones RB Shpall EJ Nawaz S Prognostic analysis of tumour angiogenesis, determined by microvessel density and expression of vascular endothelial growth factor, in high-risk primary breast cancer patients treated with high-dose chemotherapy Br J Cancer 2007 97 391 397 10.1038/sj.bjc.6603875 17609662 PMC2360317

[B29] Millanta F Silvestri G Vaselli C Citi S Pisani G Lorenzi D Poli A The role of vascular endothelial growth factor and its receptor Flk-1/KDR in promoting tumour angiogenesis in feline and canine mammary carcinomas: a preliminary study of autocrine and paracrine loops Res Vet Sci 2006 81 350 357 10.1016/j.rvsc.2006.01.007 16556453

[B30] Al-Dissi AN Haines DM Singh B Kidney BA Immunohistochemical expression of vascular endothelial growth factor receptor-2 in canine simple mammary gland adenocarcinomas Can Vet J 2010 51 1109 1111 21197202 PMC2942048

[B31] Jain RK Normalization of tumor vasculature: an emerging concept in antiangiogenic therapy Science 2005 307 58 62 10.1126/science.1104819 15637262

[B32] Lambert E Dassé E Haye B Petitfrère E TIMPs as multifacial proteins Critical Rev Oncol/Hematol 2004 49 187 198 10.1016/j.critrevonc.2003.09.008 15036259

[B33] Ree AH Florenes VA Berg JP Maelandsmo GM Nesland JM Fodstad O High levels of messenger RNAs for tissue inhibitors of metalloproteinases (TIMP-1 and TIMP-2) of primary breast carcinomas are associated with development of distant metastases Clin Cancer Res 1997 3 1623 1628 9815852

[B34] Gonzalez LO Junquera S Del Casar JM González L Marín L González-Reyes S Andicoechea A González-Fernández R González JM Pérez-Fernández R Vizoso FJ Immunohistochemical study of matrix metalloproteinases and their inhibitors in pure and mixed invasive and in situ ductal carcinomas of the breast Human Pathol 2010 41 980 989 10.1016/j.humpath.2009.08.027 20236691

[B35] Santos A Lopes C Frias C Amorim I Vicente C Gärtner F Matos A Immunohistochemical evaluation of MMP-2 and TIMP-2 in canine mammary tumours: a survival study Vet Journal2011a 190 396 402 10.1016/j.tvjl.2010.12.003 21269852

[B36] Matos AJF Faustino AMR Lopes C Rutteman GR Gärtner F Detection of lymph node micrometastases in canine malignant mammary tumours with the use of cytokeratin immunostaining Vet Record 2006 158 626 629 10.1136/vr.158.18.626 16679481

[B37] Elston CW Ellis IO Rosen PP Assessment of histological grade Rosen’s Breast Pathology 1998 1st Philadelphia: Lippincott-Raven Publ 365 382

[B38] Santos AAF Oliveira JT Lopes CCC Amorim IF Vicente CMFB Gärtner MFMR Matos AJF Immunohistochemical expression of vascular endothelial growth factor in canine mammary tumours J Comp Pathol 2010 143 268 275 10.1016/j.jcpa.2010.04.006 20570280

[B39] Santos A Lopes C Marques RM Amorim I Ribeiro J Frias C Vicente C Gärtner F Matos A Immunohistochemical analysis of urokinase plasminogen activator and its prognostic value in canine mammary tumours Vet Journal2011b 189 43 48 10.1016/j.tvjl.2010.05.023 20598596

[B40] Klein M Kleinbaum DG Gail M, Krickeberg K, Samet J, Tsiatis A, Wong W Kaplan-Meier survival curves and the Log-rank test Survival Analysis – A Self Learning Text 2005 2nd New York: Springer Science + Business Media LLC 45 82

